# Severe Sepsis in Non-Perforated Stercoral Colitis: Case Report

**DOI:** 10.51894/001c.144613

**Published:** 2025-09-30

**Authors:** Narissa Nursjamsi

**Affiliations:** 1 Department of Internal Medicine Garden City Hospital

## 106

### INTRODUCTION

Stercoral colitis (SC) is a rare complication of constipation, defined as inflammation of the colonic wall caused by increased intraluminal pressure of the colonic wall. Diagnosing and treating it in a timely manner is essential due to the high mortality rate.

### CASE REPORT

Patient was a 72 years old female with the past medical history of Hypertension, DM, Hypothyroidism, and Hyperlipidemia presented with constant, dull abdominal pain with scale of 8/10 associated with constipation. She has had a history of intermittent constipation for a few months, with the last bowel movement 3 days ago, but able to pass flatus. At the presentation, the patient’s vitals showed HR 96, RR 18, BP 137/77, and temperature 99.8 F. Her abdominal exam was benign. Unfortunately, she refused the DRE examination. Her abdominal X-ray showed constipation in the large bowel. Due to unproportionate symptoms and clinical findings, she underwent a CT abdomen, which showed fecal filled the distal sigmoid colon with mild perirectal and presacral fat stranding associated with trace fluid. Her first set of blood work showed leukocytosis 21.1, elevated lactic acid 3.02, and normal LFT, Lipase, and thyroid panel. Her blood culture was negative and no bacteria in her urinalysis. Diagnosis of sepsis secondary to non-perforated SC was established. She received IV fluid, Rocephin, Flagyl, and enema. She had a significant bowel movement after she received enema followed by Lactulose twice daily. Her clinical symptoms have resolved, and her bowel movement has gotten better. Her vitals remained stable, sepsis and stercoral colitis were successfully treated, and she was discharged without surgical intervention.

### DISCUSSION

There were no guidelines regarding diagnosis and management for SC, and cases were treated in case-by-case scenarios. In the acute setting, SC clinical symptoms were nonspecific, and CT imaging was needed for diagnosis, as in this case. Non-perforated SC with sepsis mortality was up to 63.6%, which was higher than perforated SC, 34.9%. If not treated promptly manner, stercoral colitis mortality was up to 63.6%, which was done successfully in this case and prevented mortality of the case.

